# Cardiorespiratory and Hematological Responses to High‐Intensity Interval Training in Adolescent Girls With Overweight or Obesity: A Randomized Controlled Trial

**DOI:** 10.1002/ejsc.70181

**Published:** 2026-04-28

**Authors:** Wissal Abassi, Nejmeddine Ouerghi, Moncef Feki, Santo Marsigliante, Anissa Bouassida, Beat Knechtle, Antonella Muscella

**Affiliations:** ^1^ Research Unit “Sport Sciences Health and Movement” (UR22JS01) High Institute of Sport and Physical Education of Kef University of Jendouba Kef Tunisia; ^2^ Faculty of Medicine of Tunis University of Tunis el Manar Rabta Hospital Tunis Tunisia; ^3^ University of Gafsa High Institute of Sport and Physical Education of Gafsa Gafsa Tunisia; ^4^ Department of Biological and Environmental Science and Technologies (DiSTeBA) University of Salento Lecce Italy; ^5^ Institute of Primary Care University Hospital of Zurich Zurich Switzerland; ^6^ Medbase St. Gallen Am Vadianplatz St. Gallen Switzerland

**Keywords:** adolescent girls, cardiorespiratory fitness, creatine kinase, erythrocytes, high‐intensity interval training, obesity, plasma volume variation

## Abstract

This randomized controlled trial examined the effects of a 10‐week high‐intensity interval training (HIIT) program on physiological and hematological outcomes in 28 adolescent girls with overweight or obesity. Participants were randomly assigned to a HIIT group or a control group. The HIIT protocol comprised 4 sets of 6 × 15‐s bouts at 90%–105% of maximal aerobic speed (MAS), interspersed with 15‐s active recovery at 50% MAS, performed three times per week. Significant group×time interactions were observed for body composition, aerobic capacity, maximal heart rate (HRmax), erythrocytes, hemoglobin, hematocrit, creatine kinase (CK), and lactate dehydrogenase (LDH). Between‐group comparisons showed greater improvements in body mass, body fat, waist circumference, MAS, estimated VO_2_max, and plasma volume in the HIIT group compared with controls, though not all differences reached statistical significance. Within‐group analysis in the HIIT group revealed reductions in body mass (*p* < 0.001, *d* = 0.37), body fat (*p* = 0.001, *d* = 0.78), waist circumference (*p* < 0.001, *d* = 0.54), HRmax (*p* = 0.020, *d* = 0.88), erythrocytes (*p* = 0.007, *d* = 0.46), hemoglobin (*p* = 0.019, *d* = 0.84), hematocrit (*p* = 0.004, *d* = 0.34), CK (*p* = 0.049, *d* = 0.29), and LDH (*p* = 0.032, *d* = 0.41), alongside increases in MAS (*p* = 0.007, *d* = 0.64) and estimated VO_2_max (*p* = 0.007, *d* = 0.64). Plasma volume variation was also higher in the HIIT group. Overall, a 10‐week HIIT intervention enhanced body composition, plasma volume, and cardiorespiratory fitness, while reducing markers of muscular and metabolic stress. These results indicate that HIIT is a feasible and effective approach for improving physiological and metabolic health in adolescent girls with overweight or obesity, supporting its potential as a targeted exercise strategy in this population.

## Highlights

- A 10‐week high‐intensity interval training (HIIT) program improved body composition and aerobic capacity in adolescent girls with overweight or obesity.
- HIIT significantly increased maximal aerobic speed, VO_2_max, and plasma volume compared with controls.
- Favorable hematological adaptations were observed, including reductions in hemoglobin, hematocrit, erythrocytes, CK, and LDH.
- HIIT appears to be a feasible and effective strategy to improve cardiometabolic health in adolescent girls with overweight or obesity.

## 1 Introduction

Childhood and adolescent obesity are major global health issues with long‐term consequences for cardiometabolic health (Jebeile et al. 2022). In addition to metabolic disturbances such as insulin resistance and dyslipidemia, excess body fat in youth is frequently associated with reduced cardiorespiratory fitness (Jebeile et al. 2022; Dykstra et al. 2024). Low aerobic capacity in this population has been identified as a strong and independent marker of future cardiometabolic risk, consistently linked to an increased likelihood of developing type 2 diabetes, hypertension, and cardiovascular disease later in life (Dykstra et al. 2024). Aerobic capacity is determined by a complex interplay of central and peripheral physiological factors, including maximal cardiac output, arterial oxygen content, and the efficiency of oxygen transport and utilization at the muscular level (Heinonen 2025). Hematological parameters such as hemoglobin concentration, hematocrit, red blood cell count, and plasma volume are key contributors to oxygen delivery and thus play a fundamental role in determining aerobic performance (Kanstrup and Ekblom 1982; Bekris et al. 2015).

Adolescence is marked by significant hormonal and physiological changes that influence blood volume regulation. Rising levels of estrogen and growth hormone during this period contribute to the stimulation of erythropoiesis and the expansion of plasma volume, promoting erythropoiesis and plasma volume expansion (Maughan and Shirreffs 2008). However, adolescent girls are at increased risk of iron deficiency due to rapid growth and menstrual blood loss, which can blunt hematological adaptations and reduce aerobic performance (Zimmermann and Hurrell 2007). Obesity during this stage may further disrupt blood volume regulation and hemodynamic responses to exercise (Ratchford et al. 1985; Jabbour et al. 2014). It has been associated with increased blood viscosity, systemic inflammation, and fluid imbalance, all of which may impair exercise capacity (Jabbour et al. 2014).

Additionally, chronic low‐grade inflammation and metabolic dysregulation frequently observed in adolescents with overweight or obesity may contribute to altered levels of circulating muscle enzymes. Elevated creatine kinase (CK) has been shown to associate independently with adiposity indices—such as BMI and waist circumference—even in the absence of overt muscle damage, suggesting a metabolic rather than trauma‐related origin (Haan et al. 2017). Similarly, increased CK and lactate dehydrogenase (LDH) levels have been reported in obese youth and adults, likely reflecting systemic inflammation or altered muscle metabolism rather than pathological injury (Sun et al. 2022; Bekkelund and Jorde 2018). In adolescent girls with overweight or obesity, moderate elevations in CK and LDH may therefore be indicative of subclinical metabolic stress rather than exercise‐induced muscle damage.

Moreover, studies suggest that adolescent boys with obesity may exhibit a diminished plasma volume in response to exercise training (Jabbour et al. 2014). Physical training, however, has demonstrated positive effects on body composition, aerobic fitness, and metabolic markers in youth with overweight and obesity (Ha et al. 2025).

Adolescents, particularly girls, often face barriers to engaging in traditional continuous exercise, including low motivation and time constraints (Duffey et al. 2021). High‐Intensity Interval Training (HIIT) has emerged as a time‐efficient exercise strategy that yields promising outcomes in adolescent girls with obesity, including improvements in physical fitness, insulin sensitivity, hormonal balance, inflammatory markers, and lipid profiles (Abassi et al. 2020, 2022, 2023, 2025). Despite the growing interest in interval training for youth with obesity, there is a notable lack of research examining the effects on plasma volume regulation and hematological responses in adolescent girls. Most existing studies have focused on male adults or adolescents with obesity (Jabbour et al. 2014; Jabbour et al. 2025).

The present study aims to investigate the effects of HIIT on multiple outcomes in adolescent girls with overweight or obesity, including body composition, hematological parameters, plasma volume variations, circulating muscle enzymes, and cardiorespiratory fitness. The primary outcomes were defined as cardiorespiratory and hematological responses, as these represent the main physiological adaptations expected following HIIT interventions in this population. Secondary outcomes included body composition, plasma volume, and muscle enzyme levels.

We hypothesize that HIIT is associated with improvements in cardiorespiratory fitness without causing excessive muscular or metabolic stress. To distinguish the specific effects of the HIIT intervention from normal developmental changes and external environmental influences, a non‐exercising control group was included in the study design.

## 2 Methods

### 2.1 Study Protocol

This randomized controlled trial was conducted over a 10‐week period between December 2024 and February 2025 to investigate the effects of a 10‐week HIIT program on body composition, hematological parameters, plasma volume, and cardiorespiratory fitness, including enzymes indicative of muscular and metabolic strain. Body composition parameters, including height, body mass, body mass index (BMI), body fat percentage (BF), and waist circumference (WC), were obtained with participants barefoot and wearing light clothing, following standardized procedures previously described (Abassi et al. 2020).

### 2.2 Sample Size Calculation

The sample size was estimated using G*Power software (version 3.1, Heinrich Heine University Düsseldorf, Germany). The calculation was based on detecting a group × time interaction effect for cardiorespiratory and hematological responses, which were defined as the primary outcomes of the study. An effect size of 0.55 was used, derived from a previous randomized controlled trial in obese adolescents performing HIIT interventions with a comparable design and sample size (Zhang et al. 2022; Deng and Wang 2024; Zheng et al. 2025), where significant improvements in aerobic fitness were observed. Using an alpha level of 0.05 and a statistical power of 0.80, the required minimum total sample size was 28 participants. Secondary outcomes, including body composition and muscle damage markers, were not used for the sample size calculation and should be interpreted as exploratory.

### 2.3 Participants

A total of 36 adolescent girls with overweight or obesity volunteered to participate in the study. Recruitment was carried out in public high schools located in Kalaat Sinan, in the Kef Governorate (Tunisia), through the distribution of posters and informational brochures within the schools.

All participants engaged in 3 hours of physical activity per week through the school's mandatory physical education program. Among these, two individuals were excluded for not meeting the inclusion criteria, and three withdrew prior to the intervention for personal reasons. The remaining 31 participants were randomly allocated to one of two parallel groups: a high‐intensity interval training group (HIITG; *n* = 16) or a control group (CG; *n* = 15). A stratified randomization procedure was employed based on baseline BMI and age to ensure balanced allocation across groups. The randomization sequence was generated using a computerized algorithm, with allocation concealment ensured via sealed opaque envelopes. Outcome assessors and data analysts were blinded to group assignment to minimize bias. Baseline comparisons revealed no significant differences between groups in age, anthropometric measures, or cardiorespiratory fitness (*p* > 0.05), confirming group homogeneity prior to the intervention.

**FIGURE 1:**
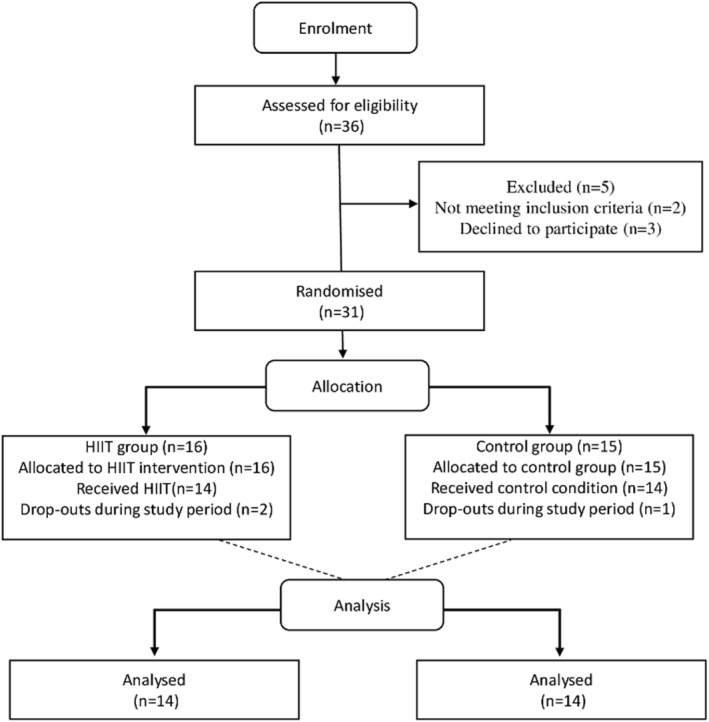
Study flow diagram. HIIT: High‐Intensity Interval Training.

During the intervention, three additional participants discontinued: two from the HIITG and one from the CG. Consequently, 28 girls completed the full study protocol (age: 16.2 ± 0.92 years; height: 1.60 ± 0.07 m; BMI: 33.3 ± 4.56 kg/m^2^), evenly distributed between groups (HIITG: *n* = 14; CG: *n* = 14) (Figure 1).

To be included, participants had to: (1) be females; (2) be aged between 15 and 17 years; (3) have a BMI greater than or equal to the 95th percentile for their age. Non‐inclusion criteria included the presence of any medical condition or pharmacological treatment that could interfere with the study protocol (e.g., hypertension, diabetes, cardiovascular, orthopedic, neuromuscular, or neurological disorders), ongoing hormone replacement therapy or obesity‐related treatments, irregular menstrual cycles, adherence to a restrictive diet or use of dietary supplements, or participation in structured physical activity within the past 3 months.

Participants were asked to maintain their usual eating habits for the duration of the study. No specific dietary recommendations or restrictions were provided. However, food intake was monitored, and nutritional status was monitored by asking participants to report any significant changes in their eating habits during the study period. Food intake was assessed using food diaries.

To monitor hydration, participants were asked to maintain their usual fluid intake throughout the study and to consume approximately 500 mL of water 2 h before all laboratory tests.

Exclusion criteria comprised withdrawal of consent, non‐compliance with study procedures, and insufficient data for analysis.

The objectives, procedures, potential risks, and benefits of the study were clearly explained to both adolescents and their legal guardians. Ethical approval was granted by Local Ethics Committee of the High Institute of Sports and Physical Education of Kef (Approval Number: ISSEPK‐0031/2024). In line with international ethical guidelines, including the Declaration of Helsinki, verbal assent was obtained from the adolescents, while written informed consent was provided by both parents or legal guardians.

All personal information will be anonymized and stored on password‐protected devices in secure facilities. Only members of the research team with appropriate clearance will have access to the data. In accordance with data protection regulations, anonymized data will be retained for 5 years and then securely destroyed.

Adherence among completers was high (97% of scheduled sessions completed), and withdrawals were unrelated to the intervention, minimizing potential bias. This approach ensures that the analysis reflects reliable and robust intervention effects.

### 2.4 Body Composition Measures

Anthropometric measurements were conducted under standardized pre‐test conditions. Participants fasted for at least 8 h, maintained usual hydration, and avoided strenuous exercise in the 24 h prior to testing. Measurements were performed at a consistent time of day to minimize diurnal variation. Anthropometric measurements were conducted according to standardized protocols, with participants wearing light clothing and no footwear. Body weight (kg) and body fat percentage were recorded using an electronic scale (Tanita BC‐533, Tokyo, Japan). Height (m) was measured using a stadiometer (Holtain Ltd., UK), and body mass index (BMI) was calculated as weight in kilograms divided by the square of height in meters (kg/m^2^). Waist circumference (cm) was measured to the nearest 0.1 cm using a non‐elastic measuring tape, placed horizontally at the midpoint between the inferior margin of the last palpable rib and the superior border of the iliac crest, with participants standing upright and breathing normally.

### 2.5 Aerobic Capacity

Maximal aerobic speed (MAS) and maximal oxygen consumption (VO_2max_) were determined using the Vameval test (Cazorla 1990). All participants completed a familiarization session with the Vameval test 1 week before baseline testing. Conducted on a 400‐m track marked every 20 m, the test begins at a running speed of 8 km/h and increases by 0.5 km/h every minute until exhaustion. This progression is dictated by audio signals that guide the runner's pace. Twenty markers are positioned at 20‐m intervals to help participants maintain the appropriate speed. The test was terminated when the participant failed to reach the marker in time for two consecutive signals.

Heart rate was monitored continuously using a Polar heart rate monitor (Polar S810, Kempele, Finland), and maximum heart rate (HR_max_) was recorded at the final stage. Although VO_2_max was estimated from field performance rather than directly measured through gas analysis, the Vameval test has shown good validity and reliability for assessing aerobic fitness in youth populations. Previous studies have reported strong associations between maximal aerobic speed obtained from the Vameval test and laboratory‐measured VO_2_max, with good test–retest reliability in adolescents (Léger and Boucher 1980; Berthoin et al. 1994; Ruiz et al. 2011). VO_2max_ values (mL/kg/min) were also expressed in metabolic equivalents (METs) by dividing by 3.5. However, because this value represents resting oxygen consumption in adults (Ainsworth et al. 2000), MET estimates in adolescents should be interpreted with caution.

### 2.6 Blood Sampling

After an overnight fast, venous blood samples were collected from each participant in the morning between 8:00 and 9:00 a.m. Participants were instructed to avoid any vigorous physical activity for 48 h prior to each blood collection to minimize the acute influence of exercise on muscle damage markers (CK and LDH).

Blood was drawn from the antecubital vein using standard aseptic techniques and collected into EDTA and lithium heparin tubes. The samples were immediately centrifuged at 3000 rpm for 10 min at 4°C, and serum was then prepared and stored at −80°C until further analysis. Erythrocytes, hematocrit, hemoglobin, mean corpuscular volume (MCV), mean corpuscular hemoglobin (MCH), and mean corpuscular hemoglobin concentration (MCHC) were analyzed with an automated hematology analyzer XN450 (Sysmex, Norderstedt, Germany). Creatine kinase (CK) and lactate dehydrogenase (LDH) levels were assessed using an automated clinical chemistry analyzer (AU480, Beckman Coulter, France).

Plasma volume variation (PVV) was estimated using the measured hematocrit (Ht) and hemoglobin (Hb) values, and the results were expressed as percentage variations (Costill and Fink 1974).

$$\%\text{PVV}=100\times \left[\left({\text{Hb}}_{A}/{\text{Hb}}_{B}\right)\times \left(100\;–\;{\text{Ht}}_{B}\right)\;/\;\left(100\;–\;{\text{Ht}}_{A}\right)\right]-1$$

where HbA and HtA denotes baseline Ht and Hb values measured prior to the training intervention, and HbB and HtB represents the corresponding post‐intervention values.

PVV represents an estimate of plasma volume changes and assumes stable red blood cell mass and hydration between measurements. This method provides a practical approach for assessing plasma volume responses in clinical populations but may be influenced by variations in hydration or iron status. Consequently, results should be interpreted with caution, and further studies using direct plasma volume measurement methods are warranted to confirm the observed adaptations.

To ensure data accuracy, post‐intervention concentrations of CK and LDH were adjusted for plasma volume variations based on these calculated values.

## 3 Data Management

Data will be electronically entered and stored on a secure research server. Independent double data entry will be performed by two team members to ensure accuracy. Built‐in range and consistency checks will be used to identify outliers and minimize data entry errors, thereby ensuring high data quality.

### 3.1 High Intensity Interval Training Program

The HIIT group followed a training protocol three times per week over 10 weeks, totaling 30 sessions. HIIT sessions included: (i) a standardized warm‐up of 5 minutes of continuous jogging at 50%MAS, followed by 5 minutes of dynamic mobility and activation drills such as high‐knee skips, high‐knee running, and butt kicks. (ii) A HIIT training session, consisting of four series of 15‐s runs at 90%–105% of the maximal aerobic speed (MAS) interspersed with 15 s of active recovery (Racil et al. 2024) (Table 1) and (iii) a cool‐down of 5 minutes of static stretching.

**TABLE 1:** Progressive high‐intensity interval training protocol over 10 weeks. MAS, maximal aerobic speed.

Training weeks	Sets per session	Repetitions per set	Run/Active recovery (s)	%MAS (run/Recovery)	Passive recovery (s)
Week 1–2	4	6	15/15	90%/50%	3
Week 3–4	4	8	15/15	90%/50%	3
Week 5–6	4	8	15/15	95%/50%	3
Week 7–8	4	8	15/15	100%/50%	3
Week 9–10	4	8	15/15	105%/50%	3

The control group continued their usual daily activities and followed the standard physical education curriculum. They were instructed not to engage in any additional structured training during the intervention period.

Session attendance was monitored, and weekly reminders were sent to participants and their parents to encourage compliance. All training sessions were supervised by qualified physical education instructors.

Adherence to the intervention was high, with participants attending more than 90% of the scheduled HIIT sessions. Participants were allowed to discontinue or modify their training in the event of adverse events (e.g., persistent musculoskeletal pain, signs of cardiovascular overload), upon personal request, or based on medical advice.

### 3.2 Statistical Analysis

Data are presented as means ± standard deviations (SD). Normality was assessed using the Shapiro–Wilk test, and homogeneity of variances was verified with Levene's test. Linear mixed models (LMM) were applied to examine the effects of the intervention, with group (HIIT vs. control) and time (pre‐vs. post‐intervention) as fixed factors, and subject ID as a random factor to account for repeated measures. Covariates including height, lean mass, and calcium intake were included to control for potential confounding effects. Group × time interactions were the primary focus. When significant interactions were detected, Bonferroni‐adjusted post hoc comparisons were performed.

To account for multiple secondary outcomes and control the risk of type I error, the False Discovery Rate (FDR) method was applied. The FDR controls the expected proportion of false positives among results declared significant, offering a balance between error control and statistical power when multiple comparisons are performed.

Effect sizes (Cohen's d) were calculated using pooled standard deviations, reported with 95% confidence intervals (CI), and interpreted as: 0.00–0.49 = small, 0.50–0.79 = moderate, ≥ 0.80 = large (Cohen 1988). Both between‐group and within‐group effect sizes were reported as appropriate.

All analyses were performed using SPSS version 27.0 (IBM Corp., Armonk, NY, USA), with significance set at *p* < 0.05.

## 4 Results

Anthropometric and biochemical data, as well as group × time interactions and eta‐squared (η^2^_p_) values gathered before and after the training program, are displayed in Table 2. There were no significant differences in these outcomes between the two groups before the interventions (*p* > 0.05).

**TABLE 2:** Evaluation of pre‐to post‐training variations in body composition, functional measures and blood‐based biomarkers among participants undergoing high‐intensity interval training (HIIT) versus controls.

	Control group (*n* = 14)		HIIT group (*n* = 14)		Interaction (time × group)		
	Pre	Post	Pre	Post	F	*P*	*η*_*p*_^2^
Age (years)	16.14 ± 0.86		16.29 ± 0.99				
Height (m)	1.60 ± 0.08		1.61 ± 0.06				
Body mass (kg)	83.4 ± 4.17	84.2 ± 4.64	85.7 ± 10.1	82.2 ± 9.81^***^	63.52	< 0.001	0.710
Body mass index (kg/m^2^)	33.1 ± 4.66	33.2 ± 4.64	33.4 ± 4.65	31.8 ± 4.61^***^	39.75	< 0.001	0.605
Body fat (%)	33.2 ± 1.53	33.4 ± 1.63	33.3 ± 1.78	32.2 ± 1.37^**^	9.626	0.005	0.270
Waist circumference (cm)	106 ± 8.80	106 ± 10.48	109 ± 10.2	104 ± 9.05^***^	8.761	0.006	0.252
Maximal aerobic speed(km/h)	9.57 ± 0.70	9.50 ± 0.71	9.29 ± 0.91	9.86 ± 0.95^**^	7.630	0.010	0.227
VO2max (mL/min/kg)	36.7 ± 2.53	36.4 ± 2.56	35.6 ± 3.31	37.7 ± 3.45^**^	7.872	0.009	0.232
HR max (bpm)	199 ± 1.87	199 ± 2.73	200 ± 1.91	197 ± 4.11^*^	4.712	0.039	0.153
Erythrocytes (103/μL)	4.58 ± 0.30	4.64 ± 0.34	4.74 ± 0.51	4.51 ± 0.52^**^	4.553	0.042	0.149
Hemoglobin (g/dL)	13.7 ± 0.76	13.9 ± 1.11	13.5 ± 1.04	12.7 ± 1.04^*^ ^#^	7.507	0.011	0.224
Hematocrit (%)	38.8 ± 3.16	39.4 ± 3.33	39.8 ± 5.97	38.0 ± 4.90**	7.112	0.013	0.215
Mean corpuscular volume (fL)	84.3 ± 5.79	84.6 ± 7.12	81.8 ± 6.61	79.9 ± 6.67	1.859	0.184	0.067
MCHC (pg)	28.3 ± 2.74	28.3 ± 2.73	27.2 ± 3.19	26.9 ± 3.80	0.375	0.545	0.014
MCH (g/d)	33.5 ± 1.51	33.7 ± 1.41	33.2 ± 1.69	32.6 ± 1.40	3.276	0.082	0.112
Creatine kinase (U/L)	115 ± 40.3	129 ± 43.9	113 ± 43.6	103 ± 30.19^*^	5.581	0.026	0.177
Lactate dehydrogenase (U/L)	222 ± 48.9	229 ± 43.4	221 ± 43.9	206 ± 31.81^*^	4.899	0.036	0.159

*Note:* Data are expressed as mean ± SD.

Abbreviations: HR max; maximum heart rate, MCHC, Mean corpuscular hemoglobin content; MHC, Mean hemoglobin concentration; VO_2_max, Maximum oxygen uptake.

^*^ *p* < 0.05.

^**^ *p* < 0.01.

^***^ *p* < 0.001: A significant difference when comparing the pre‐measure and post‐measure (time effect), separately for the 3 groups.

^#^ *p* < 0.01 (compared to post‐intervention value in the control group).

### 4.1 Anthropometric and Functional Parameters

**FIGURE 2:**
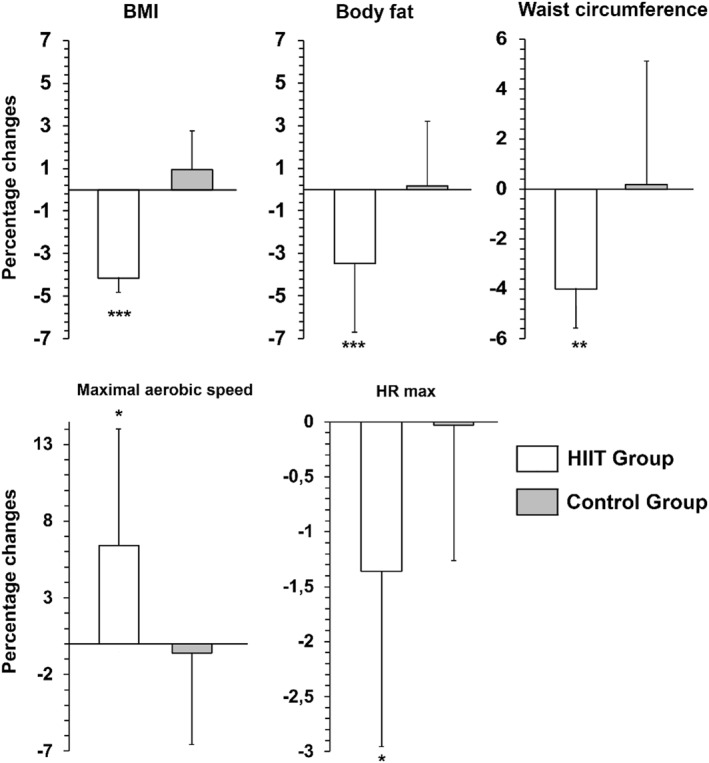
Percentage change in Body Mass Index (BMI), body fat, waist circumference, maximal aerobic speed, and maximum heart rate (HR_max_) after 10 weeks of high‐intensity interval training (HIIT) compared with a control intervention. Data are expressed as mean ± SD of the percentage change from baseline. ^*^ *p* < 0.05, ^**^ *p* < 0.01, ^***^ *p* < 0.001 versus control group.

Significant group × time interactions were observed in all anthropometric parameters, MAS, estimated VO_2max_ and HR_max_. However, post‐intervention between‐group comparisons revealed no significant differences for any of these outcomes (Table 2). Within‐group analyses indicated significant decrease in body mass (*p* < 0.001, *d* = 0.37, 95% CI [2.86 to 4.19]), BMI (*p* < 0.001, *d* = 0.35, 95% CI [1.27 to 1.87]), BF (*p* = 0.001, *d* = 0.78, 95% CI [0.57 to 1.81]), WC (*p* < 0.001, *d* = 0.54, 95% CI [3.37 to 5.48]), and HR_max_ (*p* = 0.020, *d* = 0.88, 95% CI [0.50 to 4.93]), and a significant increase in MAS (*p* = 0.007, *d* = 0.64, 95% CI [0.96 to 0.18]) and VO_2max_ (*p* = 0.007, *d* = 0.64, 95% CI [3.51 to 0.69]) after 10‐week intervention in the HIITG. No significant changes were observed in the CG (Table 2). Figure 2 shows that the changes were greater in the HIITG than in the CG for anthropometric parameters (*p* < 0.01), MAS (*p* = 0.012), VO_2max_ (*p* = 0.011) and HR_max_ (*p* = 0.039).

It should be noted that VO_2_max was estimated from the Vameval field test rather than directly measured using gas analysis; therefore, the reported values represent an indirect assessment of aerobic capacity and should be interpreted with caution.

### 4.2 Blood Biomarkers

Significant group × time interactions were observed for erythrocytes, hemoglobin, hematocrit, creatine kinase, and lactate dehydrogenase. Post‐intervention between‐group comparisons revealed significantly lower hemoglobin levels in the HIITG compared to the CG (*p* = 0.004, *d* = 1.23, 95% CI [‐2.11 to −0.44]), but no significant differences were observed for erythrocytes, hematocrit, creatine kinase, or lactate dehydrogenase (Table 2). Within‐group analyses showed significant reductions in erythrocytes (*p* = 0.007, *d* = 0.46, 95% CI [0.07 to 0.38]), hemoglobin (*p* = 0.019, *d* = 0.84, 95% CI [0.16 to 1.51]), hematocrit (*p* = 0.004, *d* = 0.34, 95% CI [0.71 to 2.90]), creatine kinase (*p* = 0.049, *d* = 0.29, 95% CI [0.08 to 21.07]), and lactate dehydrogenase (*p* = 0.032, *d* = 0.41, 95% CI [1.57 to 28.86]) in the HIITG.

**FIGURE 3:**
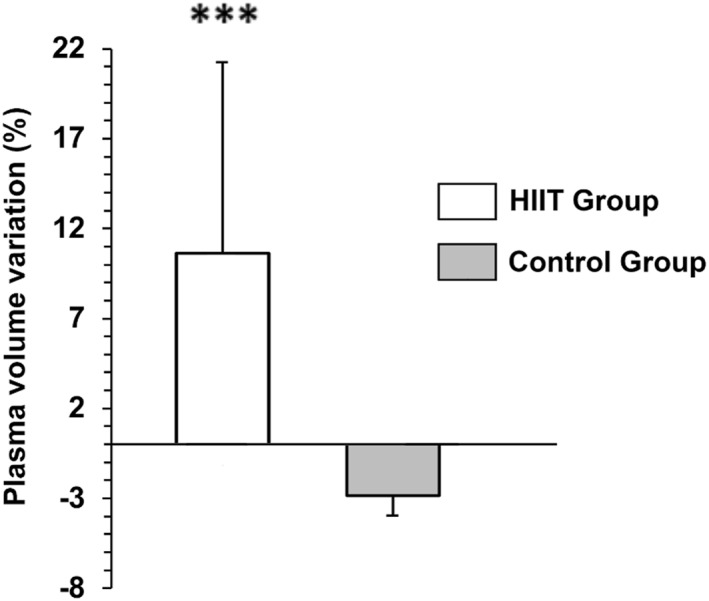
Change in plasma volume (%) after 10 weeks of high‐intensity interval training (HIIT) or in the control condition. Data are expressed as mean ± SD of the percentage change from baseline. ^***^ *p* < 0.001 versus control.

No significant time × group interactions were observed for MCV, MCHC, and MCH, and no significant between‐group differences were detected for these parameters (Table 2). PVV was significantly higher in HIITG compared to CG (10.64 ± 11.29 vs. −2.83 ± 7.36%, *p* < 0.001, *d* = 1.47, 95% CI [6.07 to 20.88]). The changes in PVV after 10 weeks under both conditions are shown in Figure 3. No significant changes were detected for any variable in the CG.

## 5 Discussion

Given their ability to detect early cardiometabolic risk, assess physical fitness, and reveal subclinical inflammation, anthropometric, functional, and hematological parameters are key clinical markers for monitoring health status and intervention efficacy in adolescents with overweight/obesity. However, little is known about how these parameters respond to high‐intensity interval training (HIIT) in this population. To the best of our knowledge, this is the first study to investigate the effects of a 10‐week HIIT program on these outcomes in adolescent girls with overweight/obesity.

Our results show that HIIT produced favorable changes in body composition (reductions in body weight and improvements in BMI, waist circumference, and body fat percentage), improved cardiorespiratory fitness, and enhanced hematological profiles. Although post‐intervention between‐group differences were not consistently significant across all variables, the direction and magnitude of changes, supported by effect sizes and confidence intervals, consistently favored the HIIT group.

In this study, the 10‐week HIIT program resulted in a significant reduction in body weight, alongside improvements in BMI, waist circumference and body fat percentage. These improvements should be interpreted in light of the interaction effects rather than solely as within‐group changes, reinforcing the effectiveness of the intervention compared to the control group.

Furthermore, the lack of statistically significant between‐group differences for some outcomes does not diminish the clinical relevance of the observed changes. Effect sizes and confidence intervals indicate meaningful improvements in the HIIT group, suggesting that the intervention may have practical benefits even when traditional significance thresholds are not met. This approach aligns with current best practices for interpreting RCT data, emphasizing interaction effects and overall trends rather than overreliance on *p*‐values alone.

These findings align with a growing body of evidence indicating that HIIT can effectively reduce body mass, total and regional fat mass, and waist circumference in adolescents with obesity (Abassi et al. 2023, 2025; Racil et al. 2023). Several systematic reviews and meta‐analyses have confirmed the efficacy of HIIT in improving body composition among this population, further supporting the clinical relevance of our results (Delgado‐Floody et al. 2019; Thivel et al. 2019).

According to clinical guidelines, a 5%–10% weight loss in adolescents with obesity is generally associated with meaningful improvements in metabolic health markers, including insulin sensitivity, blood pressure, and lipid profiles (Styne et al. 2017).These effects may be particularly relevant in adolescent girls, as hormonal fluctuations during puberty and the influence of female sex hormones, such as estrogen and progesterone, can affect fat distribution, insulin sensitivity, and energy metabolism, potentially enhancing the metabolic benefits of weight loss (Barlow 2007). Although the 4% reduction observed in our study falls slightly below this threshold, it remains clinically meaningful, especially considering the relatively short duration of the intervention. Emerging evidence suggests that even modest weight loss below can yield early metabolic benefits and facilitate long‐term behavioral changes in adolescents with obesity (Rossner 1991).

One of the primary mechanisms underlying body mass reduction following HIIT is excess post‐exercise oxygen consumption (EPOC), which significantly increases after high‐intensity exercise. EPOC elevates total energy expenditure and promotes greater fat oxidation during the recovery period, mainly through *β*‐adrenergic stimulation (Børsheim and Bahr 2003). Additionally, HIIT improves insulin sensitivity and glucose uptake in skeletal muscle, thereby enhancing metabolic efficiency and reducing fat storage (Racil et al. 2016). Hormonal modulation may also contribute, as HIIT has been shown to reduce levels of ghrelin, the hunger hormone, and increase satiety‐related hormones such as leptin, which may help decrease spontaneous energy intake (Martins et al. 2008; Abassi, Ouerghi, Muscella, et al. 2025). These hormonal effects have been documented not only in adults but also in adolescents, suggesting that HIIT may support weight management through appetite regulation during this critical developmental period (Suriano et al. 2010). This mechanism complements the increased energy expenditure induced by excess post‐exercise oxygen consumption (EPOC) and improvements in insulin sensitivity, collectively promoting favorable changes in body composition among adolescents with overweight and obesity.

Youth with overweight and obesity typically exhibit lower cardiorespiratory fitness compared to their normal‐weight peers, a condition associated with an increased risk of developing cardiovascular diseases later in life. In the present study, significant improvements were observed in cardiorespiratory fitness parameters, including maximal aerobic speed (MAS) and VO_2_max (obtained from the Vameval test), following a 10‐week HIIT intervention. A modest reduction in HRmax was also observed, which likely reflects improved pacing, greater test familiarity, or reduced anxiety during the test rather than a true physiological decrease; this change should not be interpreted as a direct physiological adaptation (Martin‐Smith et al. 2020; Deng and Wang 2024). These findings are consistent with previous research demonstrating that HIIT programs significantly improve estimated aerobic capacity in adolescents with overweight and obesity (Martin‐Smith et al. 2020; Meng et al. 2022).

HIIT significantly increased MAS and estimated VO_2_max within the intervention group, while HRmax decreased. Although these changes were statistically significant, published minimal detectable change (MDC) values for MAS and VO_2_max derived from field tests such as the Vameval test in adolescents with overweight or obesity are not available. General test–retest reliability studies indicate low day‐to‐day variability (e.g., < 5% for laboratory VO_2_max testing in adolescent girls). However, specific minimal detectable change thresholds for field‐based aerobic performance tests in obese youth remain undefined, which limits definitive interpretation relative to measurement error (Pivarnik et al. 1996).

Importantly, even modest improvements in cardiorespiratory fitness are associated with meaningful reductions in cardiovascular disease risk and premature mortality (Kodama et al. 2009). In our study, VO_2_max increased from 35.6 ± 3.31 to 37.7 ± 3.45 mL/kg/min, reflecting a small but clinically relevant improvement in estimated aerobic capacity in this population. This aligns with meta‐analyses and randomized controlled trials showing that increases of 2–4 mL·kg^−1^ min^−1^ in VO_2_max in youth with obesity are associated with reductions in blood pressure, LDL cholesterol, triglycerides, and insulin levels, alongside increases in HDL cholesterol (Zhang et al. 2022; Thivel et al. 2019; Zhu et al. 2021; Xu et al. 2025).

The observed improvements in estimated VO_2_max and MAS are likely explained by physiological adaptations induced by HIIT. Central adaptations, such as increased stroke volume, combined with peripheral adaptations, including enhanced mitochondrial enzyme activity (e.g., citrate synthase and pyruvate dehydrogenase), improve oxygen delivery to working muscles, enhance exercise tolerance, and increase cardiovascular efficiency (Zhang et al. 2022; Martin‐Smith et al. 2020; Hadjispyrou et al. 2023). Even small increases in VO_2_max are associated with measurable reductions in future cardiovascular risk, underscoring the clinical relevance of these findings (Kodama et al. 2009; Laukkanen et al. 2016).

Overall, these results support the use of HIIT as an effective strategy to improve aerobic capacity and reduce cardiometabolic risk in adolescents with overweight and obesity, even over a relatively short intervention period.

In the present study, a significant expansion of plasma volume was observed following the HIIT intervention, as evidenced by a decrease in both hematocrit and hemoglobin levels. To our knowledge, this is the first study to examine the effects of a HIIT program on plasma volume variation and related hematological markers in adolescent females with overweight/obesity.

While the observed increase in plasma volume (PV) may reflect a beneficial cardiovascular adaptation—enhancing oxygen delivery, blood flow, and reducing strain during exercise—it is important to note that PV variation (PVV) was indirectly estimated using the Dill & Costill equation, which assumes constant red cell mass, is sensitive to small analytical errors, and has not been specifically validated in obese adolescents.

Although research on PVV in adolescents with obesity is limited, evidence from adults and athletes shows that PV is highly sensitive to training intensity, duration, and participant characteristics. Highlighted the importance of fluid balance regulation in individuals with obesity, although empirical data on PV adaptations in this population remain scarce (Jabbour et al. 2014). Significant increases in plasma volume have been reported following extended HIIT interventions at intensities exceeding maximal aerobic velocity in moderately trained men (Zouhal et al. 2023; Rhibi et al. 2019), and also in untrained men and women after several weeks of low‐intensity HIIT cycling (Jabbour et al. 2018). However, some studies found no meaningful changes in PV after shorter HIIT programs in well‐trained individuals (Menz et al. 2015), reflecting variability due to training duration, fitness level, or both.

Plasma volume expansion is physiologically important because it improves stroke volume and oxygen delivery during exercise, likely contributing to the observed improvements in cardiorespiratory fitness (Convertino 1991; Coyle and González‐Alonso 2001).

These adaptations are particularly relevant in adolescent girls with obesity, as hormonal fluctuations during puberty and the influence of female sex hormones, such as estrogen and progesterone, can significantly affect fluid regulation and plasma volume variation, contributing to hematological changes observed during training or weight management interventions (Sundström Poromaa and Gingnell 2014).

Erythrocyte indices, including mean corpuscular volume (MCV), mean corpuscular hemoglobin (MCH), and mean corpuscular hemoglobin concentration (MCHC), remained largely unchanged following HIIT. Similar hematological stability has been reported in recreationally active women undergoing HIIT (Bonet et al. 2022). While these findings may suggest that reductions in hematocrit and hemoglobin could be partly attributable to plasma volume expansion, it is important to note that plasma volume was estimated indirectly. Moreover, the study population may be at risk of iron deficiency, and therefore, reductions in hematological parameters cannot be conclusively interpreted as beneficial adaptations. Alternative explanations, including transient changes in erythropoiesis or iron availability, should also be considered.

Future studies should include direct measures of plasma volume and a broader panel of hematological and iron‐related markers to better distinguish between physiological adaptations to HIIT and potential nutritional or hematological limitations. This cautious approach ensures a more accurate interpretation of hematological responses in adolescents with overweight/obesity.

In the current study, CK and LDH levels decreased after the 10‐week HIIT intervention in adolescent girls with overweight/obesity. While statistically significant, these changes were small to moderate and baseline values were within the normal range, suggesting that participants were not experiencing elevated metabolic stress prior to the intervention. Therefore, reductions in CK and LDH likely reflect physiological adaptations to regular high‐intensity exercise rather than a definitive decrease in muscle damage.

Evidence in adolescents with overweight or obesity is limited, as most research has focused on athletic or physically active populations (Du and Sim 2021; Anđelković et al. 2015). Some studies report decreases in CK and LDH after HIIT (Du and Sim 2021), while others show increases following prolonged training in team sport athletes (Anđelković et al. 2015). These discrepancies may be attributed to differences in training protocols, duration, exercise modality, and participant characteristics, particularly baseline fitness.

CK and LDH are known to vary with training status and exercise intensity (Brancaccio et al. 2010; Pilis et al. 1988). Consistent with prior evidence, high‐intensity interval training elicits adaptive responses in cardiometabolic markers (Thivel et al. 2019; Xu et al. 2025; Song and Lan 2024) and may help counteract low‐grade inflammation and cellular stress in adolescent girls with obesity, potentially contributing to the observed changes in muscle damage markers (Hertiš Petek et al. 2022; Hertiš Petek and Marčun Varda 2024; Montero et al. 2012).

Several limitations of this study should be acknowledged. First, the relatively small sample size may have limited statistical power and thus warrants cautious generalization of the findings to broader populations. Second, plasma volume was only assessed post‐intervention, precluding a direct comparison of within‐group changes over time. Third, potential confounding factors such as menstrual cycle phase, hormonal status (e.g., estrogen levels), and iron status were not controlled for, despite their known influence on hematological parameters and physiological adaptations to exercise training. Another limitation of the present study is the absence of systematic dietary monitoring throughout the intervention. Although participants were instructed to maintain their usual dietary habits and report any major changes, no quantitative assessment of energy or nutrient intake was conducted, and no weekly verification of dietary consistency was performed. Consequently, changes observed in body composition, hematological parameters, and muscle damage markers may have been partially influenced by unmeasured dietary factors. Future studies should consider including formal dietary assessment or control to better isolate the effects of HIIT interventions.

Finally, the absence of a follow‐up period limits conclusions regarding the long‐term effectiveness and sustainability of HIIT‐induced adaptations in this population. These limitations highlight the need for larger‐scale and longer‐duration studies to further validate the present findings.

## 6 Conclusion

This study shows that a 10‐week HIIT program is associated with improvements in body composition, cardiorespiratory fitness, and hematological profiles in adolescent girls with overweight/obesity. Reductions in CK and LDH levels may reflect lower subclinical metabolic stress and inflammation, although these changes should be interpreted cautiously. These findings support the potential of HIIT as an intervention that could contribute to better metabolic health and help mitigate risk factors for chronic diseases in this population. Further research is needed to confirm these effects, explore long‐term outcomes, and clarify the underlying physiological mechanisms.

## Author Contributions

Conceptualization, W.A., A.M., and N.O. methodology, W.A. software, W.A. validation, A.M., A.B., and B.K. formal analysis, W.A. investigation, W.A, resources, W.A. data curation, W.A. writing–original draft preparation, W.A. writing–review and editing, W.A., N.O.A.M., S.M., A.B., M.F., and B.K. visualization, A.B. supervision, A.B. project administration, W.A. All authors have read and agreed to the published version of the manuscript.

## Funding

This study was conducted without external funding or sponsorship. The principal investigator served as the trial sponsor.

## Ethics Statement

Ethical approval for this study was obtained from the Local Ethics Committee of the High Institute of Sports and Physical Education of Kef (Approval No. ISSEPK‐0031/2024; Approval date: September 20, 2024). In line with international ethical guidelines, including the Declaration of Helsinki, verbal assent was obtained from the adolescents, while written informed consent was provided by their parents or legal guardians. The trial was registered at ClinicalTrials.gov under the identifier: NCT07046520 (registration date: June 23, 2025). As the registration occurred after the start of participant recruitment, it should be considered retrospective.

## Conflicts of Interest

We have declared all financial and non‐financial competing interests, including funding, institutional, and personal affiliations.

## Data Availability

The data that support the findings of this study are available from the corresponding author upon reasonable request.

## References

[ejsc70181-bib-0001] Abassi, W. , N. Ouerghi , M. Feki , et al. 2023. “Effects of Moderate‐ Vs. High‐Intensity Interval Training on Physical Fitness, Enjoyment, and Affective Valence in Overweight/Obese Female Adolescents: A Pre‐/Post‐Test Study.” European Review for Medical and Pharmacological Sciences 27, no. 9: 3809–3822. 10.26355/eurrev_202305_32286. 37203805

[ejsc70181-bib-0002] Abassi, W. , N. Ouerghi , H. Ghouili , S. Haouami , and A. Bouassida . 2020. “Greater Effects of High‐ Compared With Moderate‐Intensity Interval Training on Thyroid Hormones in Overweight/Obese Adolescent Girls.” Hormone Molecular Biology and Clinical Investigation 41, no. 4: 20200031. 10.1515/hmbci-2020-0031. 33581014

[ejsc70181-bib-0003] Abassi, W. , N. Ouerghi , M. B. Hammami , et al. 2025. “High‐Intensity Interval Training Reduces Liver Enzyme Levels and Improves MASLD‐Related Biomarkers in Overweight/Obese Girls.” Nutrients 17, no. 1: 164. 10.3390/nu17010164. 39796598 PMC11723383

[ejsc70181-bib-0004] Abassi, W. , N. Ouerghi , A. Muscella , S. Marsigliante , M. Feki , and A. Bouassida . 2025. “Systematic Review: Does Exercise Training Influence Ghrelin Levels?” International Journal of Molecular Sciences 26, no. 10: 4753. 10.3390/ijms26104753. 40429895 PMC12112022

[ejsc70181-bib-0005] Abassi, W. , N. Ouerghi , P. T. Nikolaidis , et al. 2022. “Interval Training With Different Intensities in Overweight/Obese Adolescent Females.” International Journal of Sports Medicine 43, no. 5: 434–443. 10.1055/a-1648-4653. 34749418

[ejsc70181-bib-0006] Ainsworth, B. E. , W. L. Haskell , M. C. Whitt , et al. 2000. “Compendium of Physical Activities: An Update of Activity Codes and MET Intensities.” Medicine and Science in Sports and Exercise 32, no. 9: 498–504. 10.1097/00005768-200009001-00009. 10993420

[ejsc70181-bib-0007] Anđelković, M. , I. Baralić , B. Đorđević , et al. 2015. “Hematological and Biochemical Parameters in Elite Soccer Players During A Competitive Half Season.” Journal of Medical Biochemistry 34, no. 4: 460–466. 10.2478/jomb-2014-0057. 28356856 PMC4922354

[ejsc70181-bib-0008] Barlow, S. E. 2007. “Expert Committee. Expert Committee Recommendations Regarding the Prevention, Assessment, and Treatment of Child and Adolescent Overweight and Obesity: Summary Report.” Pediatrics 120, no. 4: 164–192. 10.1542/peds.2007-2329C. 18055651

[ejsc70181-bib-0009] Bekkelund, S. I. , and R. Jorde . 2018. “Creatine Kinase in Relation to Body Fat in a Caucasian Overweight and Obese Population.” Scandinavian Journal of Clinical and Laboratory Investigation 78, no. 1–2: 43–48: February–April. 10.1080/00365513.2017.1408140. 29258351

[ejsc70181-bib-0010] Bekris, E. , A. Gioldasis , I. Gissis , K. Anagnostakos , and M. Eleftherios . 2015. “From Preparation to Competitive Period in Soccer: Hematological Changes.” Sport Science Review 24, no. 1–2: 103–114. 10.1515/ssr-2015-0011.

[ejsc70181-bib-0011] Berthoin, S. , M. Gerbeaux , E. Turpin , F. Guerrin , G. Lensel‐Corbeil , and F. Vandendorpe . 1994. “Comparison of Two Field Tests to Estimate Maximum Aerobic Speed.” Journal of Sports Sciences 12, no. 4: 355–362. 10.1080/02640419408732181. 7932945

[ejsc70181-bib-0012] Bonet, J. B. , C. Javierre , J. T. Guimarães , et al. 2022. “Benefits on Hematological and Biochemical Parameters of a High‐Intensity Interval Training Program for a Half‐Marathon in Recreational Middle‐Aged Women Runners.” International Journal of Environmental Research and Public Health 19, no. 1: 498. 10.3390/ijerph19010498. 35010758 PMC8744745

[ejsc70181-bib-0013] Børsheim, E. , and R. Bahr . 2003. “Effect of Exercise Intensity, Duration and Mode on Post‐Exercise Oxygen Consumption.” Sports Medicine 33, no. 14: 1037–1060. 10.2165/00007256-200333140-00002. 14599232

[ejsc70181-bib-0014] Brancaccio, P. , G. Lippi , and N. Maffulli . 2010. “Biochemical Markers of Muscular Damage.” Clinical Chemistry and Laboratory Medicine 48, no. 6: 757–767. 10.1515/CCLM.2010.179. 20518645

[ejsc70181-bib-0015] Cazorla, G. 1990. “Proceedings of the International Symposium of Guadeloupe. Edts: Actshng and Areaps.” In Field Tests to Evaluate Aerobic Capacity and Maximal Aerobic Speed, 151–173.

[ejsc70181-bib-0016] Cohen, J. 1988. Statistical Power Analysis for the Behavioural Sciences. 2nd ed. Lawrence Erlbaum.

[ejsc70181-bib-0017] Convertino, V. A. 1991. “Blood Volume: Its Adaptation to Endurance Training.” Medicine and Science in Sports and Exercise 23, no. 12: 1338–1348. 10.1249/00005768-199112000-00004. 1798375

[ejsc70181-bib-0018] Costill, D. L. , and W. J. Fink . 1974. “Plasma Volume Changes Following Exercise and Thermal Dehydration.” Journal of Applied Physiology 37, no. 4: 521–525. 10.1152/jappl.1974.37.4.521. 4415099

[ejsc70181-bib-0019] Coyle, E. F. , and J. González‐Alonso . 2001. “Cardiovascular Drift During Prolonged Exercise: New Perspectives.” Exercise and Sport Sciences Reviews 29, no. 2: 88–92: April. 10.1097/00003677-200104000-00009. 11337829

[ejsc70181-bib-0020] Delgado‐Floody, P. , P. Latorre‐Román , D. Jerez‐Mayorga , F. Caamaño‐Navarrete , and F. García‐Pinillos . 2019. “Feasibility of Incorporating High‐Intensity Interval Training into Physical Education Programs to Improve Body Composition and Cardiorespiratory Capacity of Overweight and Obese Children: A Systematic Review.” Journal of Exercise Science and Fitness 17, no. 2: 35–40. 10.1016/j.jesf.2018.11.003. 30740131 PMC6353718

[ejsc70181-bib-0021] Deng, Y. , and X. Wang . 2024. “Effect of High‐Intensity Interval Training on Cardiorespiratory Fitness in Children and Adolescents With Overweight or Obesity: A Meta‐Analysis of Randomized Controlled Trials.” Frontiers in Public Health 12: 1269508. 10.3389/fpubh.2024.1269508. 38344230 PMC10853929

[ejsc70181-bib-0022] Du, H. , and Y. J. Sim . 2021. “Effect of Changes in Blood Fatigue Indicators, Inflammatory Markers, and Stress Hormone Levels on 100‐M Records of Sprinters Following an 8‐Week Intense Interval Training.” Journal of Exercise Rehabilitation 17, no. 5: 348–353. 10.12965/jer.2142536.268. 34805024 PMC8566107

[ejsc70181-bib-0023] Duffey, K. , A. Barbosa , S. Whiting , et al. 2021. “Barriers and Facilitators of Physical Activity Participation in Adolescent Girls: A Systematic Review of Systematic Reviews.” Frontiers in Public Health 9: 743935. 10.3389/fpubh.2021.743935. 34722450 PMC8553996

[ejsc70181-bib-0024] Dykstra, B. J. , G. J. Griffith , M. S. Renfrow , A. D. Mahon , and M. P. Harber . 2024. “Cardiorespiratory and Muscular Fitness in Children and Adolescents With Obesity.” Current Cardiology Reports 26, no. 5: 349–357. 10.1007/s11886-024-02036-3. 38460068

[ejsc70181-bib-0025] Ha, M. S. , H. Y. Moon , M. Lee , and J. S. Yook . 2025. “Exercise Improves Body Composition, Physical Fitness, and Blood Levels of C‐Peptide and IGF‐1 in 11‐ to 12‐Year‐Old Boys With Obesity.” Frontiers in Physiology 15: 1451427. 10.3389/fphys.2024.1451427. 39822775 PMC11735414

[ejsc70181-bib-0026] Haan, Y. C. , I. Oudman , F. S. Diemer , et al. 2017. “Creatine Kinase as a Marker of Obesity in a Multi‐Ethnic Population.” Molecular and Cellular Endocrinology 442: 24–31: February. 10.1016/j.mce.2016.11.022. 27894867

[ejsc70181-bib-0027] Hadjispyrou, S. , P. C. Dinas , S. M. Delitheos , I. A. Koumprentziotis , C. Chryssanthopoulos , and A. Philippou . 2023. “The Effect of high‐intensity Interval Training on Mitochondrial Associated Indices in Overweight and Obese Adults: A Systematic Review and Meta‐Analysis.” Frontiers in Bioscience (Landmark Edition) 28, no. 11: 281. 10.31083/j.fbl2811281. 38062841

[ejsc70181-bib-0028] Heinonen, I. 2025. “Cardiac Output Limits Maximal Oxygen Consumption, But What Limits Maximal Cardiac Output?” Experimental Physiology 110, no. 5: 666–674. 10.1113/EP091594. 40193294 PMC12053887

[ejsc70181-bib-0029] Hertiš Petek, T. , and N. Marčun Varda . 2024. “Childhood Cardiovascular Health, Obesity, and Some Related Disorders: Insights Into Chronic Inflammation and Oxidative Stress.” International Journal of Molecular Sciences 25, no. 17: 9706. 10.3390/ijms25179706. 39273654 PMC11396019

[ejsc70181-bib-0030] Hertiš Petek, T. , T. Petek , M. Močnik , and N. Marčun Varda . 2022. “Systemic Inflammation, Oxidative Stress and Cardiovascular Health in Children and Adolescents: A Systematic Review.” Antioxidants 11, no. 5: 894. 10.3390/antiox11050894. 35624760 PMC9137597

[ejsc70181-bib-0031] Jabbour, G. , D. Curnier , S. Lemoine‐Morel , R. Jabbour , M. È Mathieu , and H. Zouhal . 2014. “Plasma Volume Variation With Exercise: A Crucial Consideration for Obese Adolescent Boys.” Applied Physiology, Nutrition and Metabolism 39, no. 1: 95–100. 10.1139/apnm-2012-0493. 24383512

[ejsc70181-bib-0032] Jabbour, G. , H. D. Iancu , H. Zouhal , P. Mauriège , D. R. Joanisse , and L. J. Martin . 2018. “High‐Intensity Interval Training Improves Acute Plasma Volume Responses to Exercise That Is Age Dependent.” Physics Reports 6, no. 4: e13609. 10.14814/phy2.13609. PMC5820462 29464883

[ejsc70181-bib-0033] Jabbour, G. , M. Sellami , and H. D. Iancu . 2025. “Plasma Volume Variations in Response to High Intensity Interval Training in Obese Women: The Influential Role of Menopausal Status and Age.” Experimental Gerontology 199: 112664. 10.1016/j.exger.2024.112664. 39701433

[ejsc70181-bib-0034] Jebeile, H. , A. S. Kelly , G. O'Malley , and L. A. Baur . 2022. “Obesity in Children and Adolescents: Epidemiology, Causes, Assessment, and Management.” Lancet Diabetes and Endocrinology 10, no. 5: 351–365. 10.1016/S2213-8587(22)00047-X. 35248172 PMC9831747

[ejsc70181-bib-0035] Kanstrup, I. L. , and B. Ekblom . 1982. “Acute Hypervolemia, Cardiac Performance, and Aerobic Power During Exercise.” Journal of Applied Physiology: Respiratory, Environmental and Exercise Physiology 52, no. 5: 1186–1191. 10.1152/jappl.1982.52.5.1186. 7096143

[ejsc70181-bib-0036] Kodama, S. , K. Saito , S. Tanaka , et al. 2009. “Cardiorespiratory Fitness as a Quantitative Predictor of All‐Cause Mortality and Cardiovascular Events in Healthy Men and Women: A meta‐analysis.” JAMA 301, no. 19: 2024–2035. 10.1001/jama.2009.681. 19454641

[ejsc70181-bib-0037] Laukkanen, J. A. , F. Zaccardi , H. Khan , S. Kurl , S. Y. Jae , and R. Rauramaa . 2016. “Long‐Term Change in Cardiorespiratory Fitness and All‐Cause Mortality: A Population‐Based Follow‐Up Study.” Mayo Clinic Proceedings 91, no. 9: 1183–1188. 10.1016/j.mayocp.2016.05.014. 27444976

[ejsc70181-bib-0038] Léger, L. , and R. Boucher . 1980. “An Indirect Continuous Running Multistage Field Test: The Université De Montréal Track Test.” Canadian Journal of Applied Sport Sciences 5, no. 2: 77–84. PMID: 7389053. 7389053

[ejsc70181-bib-0039] Martins, C. , L. Morgan , and H. Truby . 2008. “A Review of the Effects of Exercise on Appetite Regulation: An Obesity Perspective.” International Journal of Obesity 32, no. 9: 1337–1347. 10.1038/ijo.2008.98. 18607378

[ejsc70181-bib-0040] Martin‐Smith, R. , A. Cox , D. S. Buchan , J. S. Baker , F. Grace , and N. Sculthorpe . 2020. “High Intensity; Interval Training (HIIT) Improves Cardiorespiratory Fitness (CRF) in Healthy, Overweight and Obese Adolescents: A Systematic Review and Meta‐Analysis of Controlled Studies.” Int J Environ Res Public Health 17, no. 8: 2955. 10.3390/ijerph17082955. 32344773 PMC7215828

[ejsc70181-bib-0041] Maughan, R. J. , and S. M. Shirreffs . 2008. “Development of Individual Hydration Strategies for Athletes.” International Journal of Sport Nutrition and Exercise Metabolism 18, no. 5: 457–472. 10.1123/ijsnem.18.5.457. 19033609

[ejsc70181-bib-0042] Meng, C. , T. Yucheng , L. Shu , and Z. Yu . 2022. “Effects of School‐Based High‐Intensity Interval Training on Body Composition, Cardiorespiratory Fitness and Cardiometabolic Markers in Adolescent Boys With Obesity: A Randomized Controlled Trial.” BMC Pediatrics 22, no. 1: 112. 10.1186/s12887-021-03079-z. 35232402 PMC8886768

[ejsc70181-bib-0043] Menz, V. , J. Strobl , M. Faulhaber , H. Gatterer , and M. Burtscher . 2015. “Effect of 3‐Week High‐Intensity Interval Training on VO2max, Total Haemoglobin Mass, Plasma and Blood Volume in Well‐Trained Athletes.” European Journal of Applied Physiology 115, no. 11: 2349–2356. 10.1007/s00421-015-3211-z. 26164709

[ejsc70181-bib-0044] Montero, D. , G. Walther , A. Perez‐Martin , E. Roche , and A. Vinet . 2012. “Endothelial Dysfunction, Inflammation, and Oxidative Stress in Obese Children and Adolescents: Markers and Effect of Lifestyle Intervention.” Obesity Reviews 13, no. 5: 441–455. 10.1111/j.1467-789X.2011.00956.x. 22133012

[ejsc70181-bib-0045] Pilis, W. , J. Langfort , A. Pilsniak , M. Pyzik , and M. Btasiak . 1988. “Plasma Lactate Dehydrogenase and Creatine Kinase After Anaerobic Exercise.” Int J Sports Med 9, no. 2: 102–103. 10.1055/s-2007-1024987. 3384513

[ejsc70181-bib-0046] Pivarnik, J. M. , M. C. Dwyer , and M. A. Lauderdale . 1996. “The Reliability of Aerobic Capacity (Vo2Max) Testing in Adolescent Girls.” Research Quarterly for Exercise and Sport 67, no. 3: 345–348. 10.1080/02701367.1996.10607962. 8888423

[ejsc70181-bib-0047] Racil, G. , C. Aouichaoui , A. Hawani , et al. 2024. “The Impact of Interval Training on Adiponectin to Leptin Ratios and on Blood Pressures in Severely Obese Adolescent Girls: A Randomized Controlled Trial.” Journal of Sports Sciences 42, no. 10: 1–9. 10.1080/02640414.2024.2369447. 38904424

[ejsc70181-bib-0048] Racil, G. , J. B. Coquart , W. Elmontassar , et al. 2016. “Greater Effects of High‐ Compared With Moderate‐Intensity Interval Training on Cardio‐Metabolic Variables, Blood Leptin Concentration and Ratings of Perceived Exertion in Obese Adolescent Females.” Biology of Sport 33, no. 2: 145–152. 10.5604/20831862.1198633. 27274107 PMC4885625

[ejsc70181-bib-0049] Racil, G. , L. Russo , G. M. Migliaccio , et al. 2023. “High‐Intensity Interval Training in Female Adolescents With Moderate or Severe Obesity.” Children 10, no. 9: 1495. 10.3390/children10091495. 37761456 PMC10528164

[ejsc70181-bib-0050] Ratchford, S. M. , J. F. Lee , K. Bunsawat , et al. 1985. “The Impact of Obesity on the Regulation of Muscle Blood Flow During Exercise in Patients With Heart Failure With a Preserved Ejection Fraction.” Journal of Applied Physiology 132, no. 5: 1240–1249. 10.1152/japplphysiol.00833.2021. PMC9126213 35421322

[ejsc70181-bib-0051] Rhibi, F. , J. Prioux , M. B. Attia , A. C. Hackney , H. Zouhal , and A. B. Abderrahman . 2019. “Increase Interval Training Intensity Improves Plasma Volume Variations and Aerobic Performances in Response to Intermittent Exercise.” Physiology and Behavior 199: 137–145. 10.1016/j.physbeh.2018.11.020. 30458187

[ejsc70181-bib-0052] Rossner, S. 1991. “Factors Determining the Long‐Term Outcome of Obesity Treatment.” In Obesity, edited by P. Bjorntorp and B. N. Brodoff , 712–719. J.B. Lippincott Co;.

[ejsc70181-bib-0053] Ruiz, J. R. , J. Castro‐Piñero , V. España‐Romero , et al. 2011. “Field‐Based Fitness Assessment in Young People: The ALPHA Health‐Related Fitness Test Battery for Children and Adolescents.” British Journal of Sports Medicine 45, no. 6: 518–524. 10.1136/bjsm.2010.075341. 20961915

[ejsc70181-bib-0054] Song, Y. , and H. Lan . 2024. “The Effects of High‐Intensity Interval Training on Cardiometabolic Health in Children and Adolescents: A Systematic Review and Meta‐Analysis.” Journal of Sports Science and Medicine 23, no. 4: 690–706. 10.52082/jssm.2024.690. 39649559 PMC11622044

[ejsc70181-bib-0055] Styne, D. M. , S. A. Arslanian , E. L. Connor , et al. 2017. “Pediatric Obesity‐Assessment, Treatment, and Prevention: An Endocrine Society Clinical Practice Guideline.” Journal of Cinical Endocrinology and Metabolism 102, no. 3: 709–757. 10.1210/jc.2016-2573. PMC6283429 28359099

[ejsc70181-bib-0056] Sun, J. , L. Wang , Y. Lin , et al. 2022. “Anthropometric Parameters of Obesity Can be Alternative Biomarkers for the Potential Cardiac Dysfunction in Obese Children.” Frontiers in Cardiovascular Medicine 9: 850071: August. 10.3389/fcvm.2022.850071. 36061547 PMC9436000

[ejsc70181-bib-0057] Sundström Poromaa, I. , and M. Gingnell . 2014. “Menstrual Cycle Influence on Cognitive Function and Emotion Processing‐From a Reproductive Perspective.” Frontiers in Neuroscience 8: 380: November. 10.3389/fnins.2014.00380. 25505380 PMC4241821

[ejsc70181-bib-0058] Suriano, K. , J. Curran , S. M. Byrne , T. W. Jones , and E. A. Davis . 2010. “Fatness, Fitness, and Increased Cardiovascular Risk in Young Children.” Journal of Pediatrics 157, no. 4: 552–558. 10.1016/j.jpeds.2010.04.042. 20542285

[ejsc70181-bib-0059] Thivel, D. , J. Masurier , G. Baquet , et al. 2019. “High‐Intensity Interval Training in Overweight and Obese Children and Adolescents: Systematic Review and Meta‐Analysis.” Journal of Sports Medicine and Physical Fitness 59, no. 2: 310–324. 10.23736/S0022-4707.18.08075-1. 29589408

[ejsc70181-bib-0060] Xu, G. , Q. Li , Q. Yang , and H. Yu . 2025. “The Effect of High‐Intensity Interval Training on Health‐Related Outcomes in Obese Adolescents: A Systematic Review and Meta‐Analysis.” Frontiers in Physiology 16: 1609818. 10.3389/fphys.2025.1609818. 40904707 PMC12403219

[ejsc70181-bib-0061] Zhang, L. , D. Wang , S. Liu , F. F. Ren , L. Chi , and C. Xie . 2022. “Effects of Acute High‐Intensity Interval Exercise and High‐Intensity Continuous Exercise on Inhibitory Function of Overweight and Obese Children.” Int J Environ Res Public Health 19, no. 16: 10401. 10.3390/ijerph191610401. 36012036 PMC9408170

[ejsc70181-bib-0062] Zheng, W. , M. Yin , Y. Guo , et al. 2025. “Effects and Moderators of High‐Intensity Interval Training and Moderate‐Intensity Continuous Training Among Children and Adolescents With Overweight or Obesity: A Systematic Review and Meta‐Analysis.” Frontiers in Physiology 16: 1625516. 10.3389/fphys.2025.1625516. 40809289 PMC12343602

[ejsc70181-bib-0063] Zhu, L. , J. Liu , Y. Yu , and Z. Tian . 2021. “Effect of High‐Intensity Interval Training on Cardiometabolic Risk Factors in Childhood Obesity: A Meta‐Analysis.” Journal of Sports Medicine and Physical Fitness 61, no. 5: 743–752. 10.23736/S0022-4707.20.11329-X. 33975429

[ejsc70181-bib-0064] Zimmermann, M. B. , and R. F. Hurrell . 2007. “Nutritional Iron Deficiency.” Lancet 370, no. 9586: 511–520. 10.1016/S0140-6736(07)61235-5. 17693180

[ejsc70181-bib-0065] Zouhal, H. , F. Rhibi , A. Salhi , et al. 2023. “The Effects of Exercise Training on Plasma Volume Variations: A Systematic Review.” International Journal of Sports Medicine 44, no. 6: 406–419. 10.1055/a-1667-6624. 34638157

